# Acute Effect of Pore-Forming *Clostridium perfringens* ε-Toxin on Compound Action Potentials of Optic Nerve of Mouse

**DOI:** 10.1523/ENEURO.0051-17.2017

**Published:** 2017-08-10

**Authors:** Mercè Cases, Artur Llobet, Beatrice Terni, Inmaculada Gómez de Aranda, Marta Blanch, Briain Doohan, Alexander Revill, Angus M. Brown, Juan Blasi, Carles Solsona

**Affiliations:** 1 Laboratory of Cellular and Molecular Neurobiology, Department of Pathology and Experimental Therapeutics, Faculty of Medicine and Health Sciences, Campus of Bellvitge, University of Barcelona, Hospitalet de Llobregat, Barcelona 08907, Spain; 2 Institut d’Investigació Biomèdica de Bellvitge - Biomedical Research Institute of Bellvitge (IDIBELL), Hospitalet de Llobregat, Barcelona 08907, Spain; 3 Institute of Neurosciences, University of Barcelona, Barcelona 08035, Spain; 4 School of Biomedical Sciences, Queen’s Medical Centre, University of Nottingham, Nottingham NG7 2UH, United Kingdom; 5 Department of Neurology, University of Washington, Seattle, WA 98195

**Keywords:** action potential, ATP release, clostridial toxin, electrophysiology, myelin, optic nerve

## Abstract

ε-Toxin is a pore forming toxin produced by *Clostridium perfringens* types B and D. It is synthesized as a less active prototoxin form that becomes fully active upon proteolytic activation. The toxin produces highly lethal enterotoxaemia in ruminants, has the ability to cross the blood–brain barrier (BBB) and specifically binds to myelinated fibers. We discovered that the toxin induced a release of ATP from isolated mice optic nerves, which are composed of myelinated fibers that are extended from the central nervous system. We also investigated the effect of the toxin on compound action potentials (CAPs) in isolated mice optic nerves. When nerves were stimulated at 100 Hz during 200 ms, the decrease of the amplitude and the area of the CAPs was attenuated in the presence of ε-toxin. The computational modelling of myelinated fibers of mouse optic nerve revealed that the experimental results can be mimicked by an increase of the conductance of myelin and agrees with the pore forming activity of the toxin which binds to myelin and could drill it by making pores. The intimate ultrastructure of myelin was not modified during the periods of time investigated. In summary, the acute action of the toxin produces a subtle functional impact on the propagation of the nerve action potential in myelinated fibers of the central nervous system with an eventual desynchronization of the information. These results may agree with the hypothesis that the toxin could be an environmental trigger of multiple sclerosis (MS).

## Visual Abstract

**Figure:**
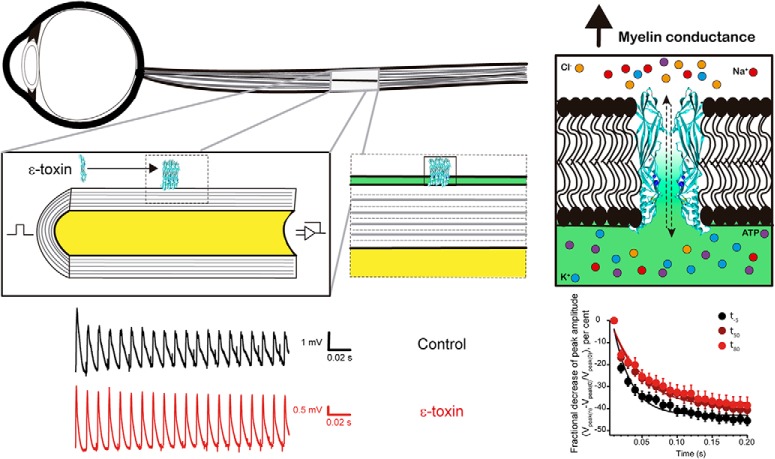

## Significance Statement

Multiple sclerosis (MS) is an inflammatory demyelinating disease of an unknown etiology. Myelin is the lipid wrap that sheathes the axons of neurons and ensures high speed of communication between the different parts of the brain. The ε-toxin has been involved recently as a possible cause of MS. Although ε-toxin induced lesions in the central nervous system have been described, the effect on the neuronal conductivity is unknown. Our study using optic nerves of mice explains the action of the toxin on brain. The finding that the toxin acutely permeabilizes myelin, induces the release of ATP and modifies the repetitive compound action potentials (CAPs) of nerves can determine its first molecular steps in relation of the disease.

## Introduction

*Clostridium perfringens*, an anaerobic Gram-positive bacterium, can produce >16 toxins (Uzal et al., 2015). According to toxin production, this microorganism is classified into five toxinotypes, A to E. Types B and D synthesize ε-toxin, responsible for a highly mortal enterotoxaemia in sheep, goats and other ruminants. Genetics, structural biology and the use of several animal models, gave some clues on the action of the toxin (Uzal et al., 2015; Freedman et al., 2016). The ε-toxin is one of the most lethal bacterial toxins known, just below botulinum and tetanus toxin (Popoff and Poulain, 2010; Alves et al., 2014). The toxin is produced as a less toxic precursor molecule (ε-prototoxin) that is activated by proteolytic cleavage of amino and carboxy terminals.

The molecular structure of the toxin was resolved using x-ray crystallography, indicating that it is an elongated protein of 100 Å in its long axe with three domains consisting of β-sheets (Alves et al., 2014). Interestingly ε-toxin shares the structural domains of pore-forming toxins, like aerolysin (Petit et al., 2001). When oriented on its *y*-axis, the upper part of the molecule configures the Domain I, which contains a large α-helix followed by loop and the β-sheet. Experimental work indicates that Domain I participates in the recognition and binding to specific receptors, while Domains II and III participate in oligomerization of the toxin and the formation of a pore in the plasma membrane of target cells (Alves et al., 2014).

ε-Toxin preferentially accumulates in brain and kidney of experimentally intoxicated mice where it exerts an acute effect (Nagahama and Sakurai, 1991; Finnie, 2004; Soler-Jover et al., 2004). In injected mice, the toxin shows the capacity to cross the blood–brain barrier (BBB), entering the brain parenchyma (Dorca-Arévalo et al., 2008). Other studies show that ε-toxin binds to components of synaptosomal fractions (Soler-Jover et al., 2007), lipid rafts (Gil et al., 2015), myelinated structures (Dorca-Arévalo et al., 2008; Wioland et al., 2015) and oligodendrocytes (Lonchamp et al., 2010; Linden et al., 2015; Wioland et al., 2015). Moreover, it was considered as a cause of demyelination measured as the decrease of MBP (Myelin Basic Protein) immunoreactivity after >20 h of toxin exposure (Linden et al., 2015; Wioland et al., 2015).

Recently, it has been speculated that ε-toxin may be an agent that elicits multiple sclerosis (MS) (Rumah et al., 2013). The hypothetical participation of ε-toxin in the etiology of MS is supported by the finding that *C. perfringens* type B was isolated from a patient at the initial stage of the disease (Rumah et al., 2013) and by the observation that ε-toxin crosses the BBB and binds to some specific components of myelin (Dorca-Arévalo et al., 2008; Rumah et al., 2015). Besides this hypothesis, little is known about the acute action of ε-toxin on myelinated fibers of the central nervous system.

In the present study, we want to uncover the possible effect of ε-toxin over the action potentials of myelinated fibers. We used mice optic nerves as a model for the activity of myelin sheaths of the central nervous system and investigated the modification in the functionality and structure of the optic nerve when exposed to ε-toxin. We studied ATP release and electrophysiological effects on compound action potentials (CAPs) of mice optic nerves. We combined low and high stimulation rates to set the optic nerve in non-stressful or stressful conditions. Moreover, we analyzed the ultrastructure of the optic nerve to detect a possible structural modification. All of our results demonstrated that the toxin causes ATP release and has an effect on trains of action potentials.

## Materials and Methods

### Expression of cDNA constructs of ε-prototoxin and ε-toxin

Based on a previously described plasmid containing the cDNA for the ε-prototoxin (Soler-Jover et al., 2004), we generated an expression vector to produce a recombinant protein with a 6 Histidine tag at the ε-prototoxin C terminal. The expression vector encoding ε-prototoxin was transformed into a RossetaTM(DE3)pLysS *Escherichia coli* strain for optimum protein expression. The expression of ε-prototoxin recombinant protein was induced overnight at room temperature in 250-ml LB medium cultures containing 1mM isopropyl-β-D-thiogalactopyranoside. Cells were pelleted and resuspended in ice cold phosphate buffer, 20 mM, pH 7.4, containing 250 mM NaCl (PBS) and were sonicated and then centrifuged at 15,000 × *g* for 20 min at 4°C. The resulting supernatant was incubated with 0.5 ml of previously equilibrated Ni-agarose beads (Talon) washed with PBS and eluted with PBS containing 250 mM Imidazol. After dialyze the eluate with PBS to eliminate Imidazol, protein content was quantified, analyzed by SDS-PAGE and stored at -20°C, until used. Full active ε-toxin was obtained by trypsin proteolysis of ε-prototoxin, using trypsin beads (Sigma-Aldrich), according to the manufacturer’s instructions. The toxicity of prototoxin and activated toxin was tested in MDCK cells as described elsewhere (Soler-Jover et al., 2004). The process of purification was performed following the guidelines of biosecurity of the University of Barcelona.

### ATP release measurement

ATP release from optic nerves was measured using a Luciferase and D-luciferin mixture. Luciferase extract lantern from Photinus pyralis (Sigma-Aldrich) was resuspended at a concentration of 0.1 µg/µl and desalted into a 10-ml 10 DG column (Bio-Rad). D-luciferin (Sigma-Aldrich) was diluted at a concentration of 0.7 µg/µl in ultrapure water and the pH adjusted to 7.4 with NaOH. A volume of 500 µl of Locke solution was placed into a test tube with 5 µl of D-luciferin and 5 µl of luciferase. In each experiment, 8-10 optic nerves were immersed into the tube. Light emitted when ATP reacted with luciferin and luciferase was captured by a photomultiplier (Hamamatsu R374) fed at high voltage (1000 V; Hamamatsu C9525 High Voltage Power Supply). The resulting signal was amplified 200× in a LPF-100B amplifier (Warner Instruments) connected in series with 902 filter (Frequency devices) at a rate of 100 Hz. The signal was digitized via USB 6341 card (National Instruments) into a computer where the signal was acquired and stored using the WinWCP (v4.0.0.8) software Strathclyde University, Scotland, United Kingdom (RRID:SCR_014713).

### Electrophysiology

**Figure 1.**
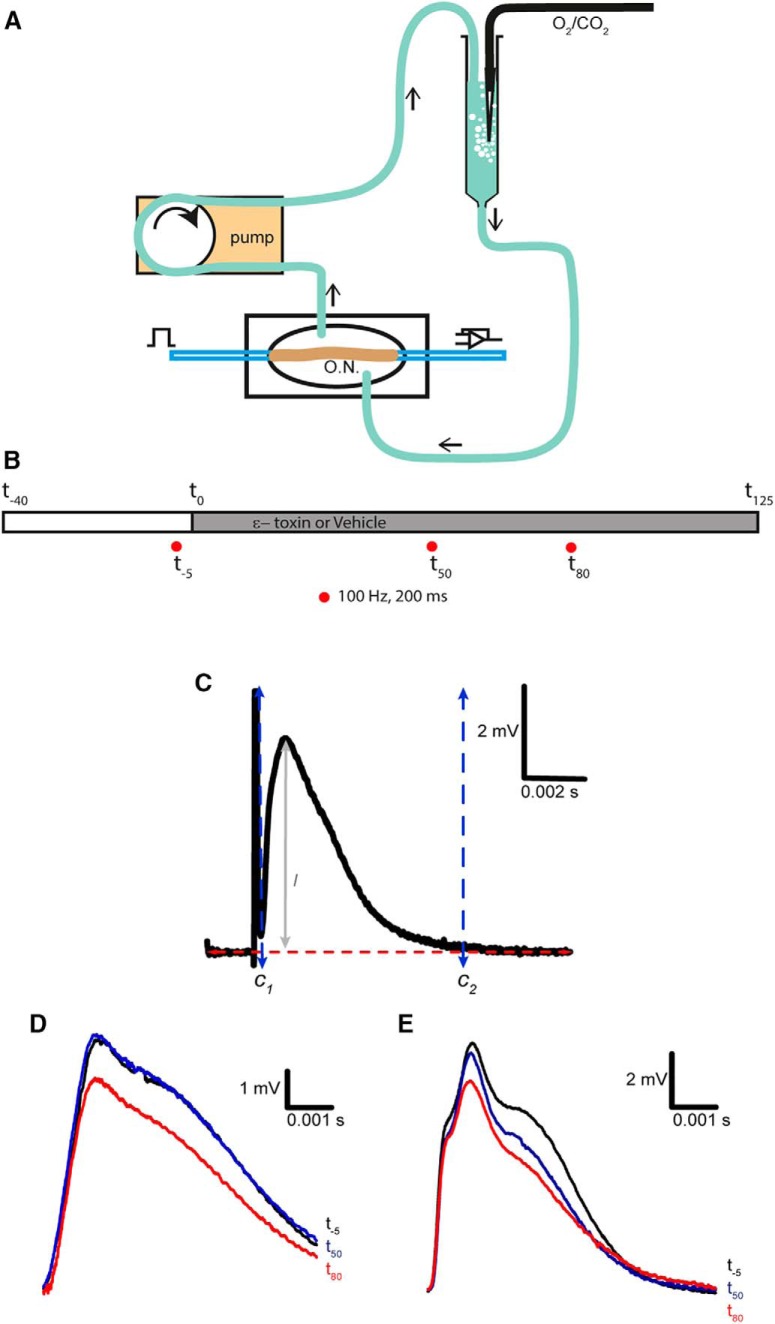
Recording CAPs from mouse optic nerve. ***A***, Set-up diagram, the signal is generated with a stimulator and conveyed into the optic nerve through a suction electrode, then recorded using a second suction electrode and send to the amplifier. At the same time, the recording chamber is perfused with Locke solution, which recirculates to get aerated in carbogen every turn. O.N., optic nerve. ***B***, Time line of experiment. The time was set at 0 once the ε-toxin is added to the recording chamber. Three train stimulations of 100 Hz that lasted for 200 ms were applied to the optic nerve: one before the addition of the ε-toxin (*t*_-5_), 50 min later (*t*_50_), and a last, 80 min (*t*_80_). Before ε-toxin addition, the optic nerve is let 30 min to stabilize to the set-up and Locke solution. ***C***, Settings for CAPs analysis. The axes were placed to calculate individually the amplitude and area of the CAP. C_1_ and C_2_ are the axes set at the beginning and at the end of the CAP, respectively. The red line is set at the base level. *l* determines the maximum amplitude of the CAP. ***D***, CAPs elicited at low-frequency stimulation (0.03 Hz) in control conditions before and after adding 50 µl of PBS (vehicle). At -5 min (black), minute 50 (blue), and 85 min (red). ***E***, In ε-toxin condition, examples of CAPs at the same given times. ε-toxin was added dissolved in PBS. Scale bars are represented in each panel. Stimulus artifact was eliminated manually. Differences in shape and amplitudes of initial CAPs in ***D***, ***E*** (black) are not related to the action of the ε-toxin, they represent the variability of recording CAPs of different animals.

Recordings were made from optic nerves of C57BL/6J male mice (RRID:IMSR_JAX:000664) of 8-12 weeks of age (Envigo) at 25-30° C. In the animal core facility, mice were kept under conventional conditions in acclimatized rooms with free access to standard pelleted food and tap water. All animal procedures were performed in accordance with regulations of the University of Barcelona animal core committee. Mice were sacrificed through cervical dislocation or CO_2_. Eye balls were pulled out and extracted from the mouse, afterward the optic nerve was dissected out from eye ball and optic muscles. The nerves were cleaned from dural sheets and placed in a recording chamber and perfused with artificial cerebrospinal fluid (Locke solution) containing: 136 mM NaCl, 5.6 mM KCl, 14.3 mM NaHCO_3_, 1.2 mM NaH_2_PO_4_, 2.2 mM CaCl_2_, 1.2 mM MgCl_2_, and 11 mM dextrose, equilibrated continuously with 95% O_2_, 5% CO_2_, pH 7.2-7.4 (Slater et al., 2013). The chamber was continuously perfused by recirculating Locke solution aerated by a gas mixture of 95% O_2_/5% CO_2_ every round. The total volume of recirculating liquid was 7 ml. Locke solution was recirculated by the use of a peristaltic pump. Nerves were allowed to equilibrate in Locke solution for 30 min before beginning the experiment. Suction electrodes back filled with Locke solution were used for stimulation and recording (Fig. 1*A*). During the experiment, the CAP was elicited orthodromically, from the rostral end, every 30 s or by 200 ms 100 Hz train stimulations (S48 Stimulator and a stimulus isolation unit 5B, Grass). Supramaximal stimuli of 50µs duration were applied. The elicited signal was amplified 100× and filtered at 33 kHz by a Grass p16 differential amplifier. The exiting signals were passed through 50/60 Hz eliminator (Quest Scientific) to obtain stable record with a noise below 100 μV. The CAPs were digitized via a USB 6341 card (National Instruments) and stored in a personal computer. Data acquisition and storage was controlled with WinWCP (V.4.0.8) software. The frequency of acquisition was 3.84 kHz. In every experiment, the CAPs were only stored when reached a constant amplitude (usually after 30 min of 0.03 Hz stimulation frequency). The time in which ε-toxin was added was considered time 0, at a final concentration of 200 nM, this is a supramaximal lethal dose concentration in MDCK cells (Soler-Jover et al., 2004). Three train stimulations were conducted at -5minutes (*t_-5_*), 50 min (*t*_50_) and 80 min (*t*_80_), respectively, from toxin incorporation (Fig. 1*B*). In some experiments, at minute 115, 100 μM 3,4-diaminopyridine (3,4-DAP) was added to block Kv channels. The final concentration was reached by adding a small volume of a stock solution of 50 mM 3,4-DAP that was prepared in Locke solution and bubbled for at least 30 min to give a pH 7.5-7.6.

The peak amplitude and area of CAPs at 0.03 or 100 Hz were measured individually using the Analysis tool from WinWCP. The axes to determine the amplitude and area were set at the baseline of the signal and vertically the two axes were fixed before and after the CAP. The axis before the CAP was set to the minimum point between the stimulus artifact and the CAP response (Fig. 1*C*). The axis after the CAP was set to the point where the CAP response goes back to the base level (Fig. 1*C*). On the other hand, the latency was measured automatically by Igor software (RRID:SCR_000325) using a macro made in the laboratory which calculated the time from the minimum base level between the stimulus artifact and the CAP response to the maximum value point of the CAP. Digitized data of the CAPs were transferred to files (Microsoft Excel or Igor).

### Computer simulations

Computer simulations were conducted with NEURON 7.1 software (Hines and Carnevale, 1997; RRID:SCR_005393). The cell-builder module was used to construct a model consisting of a soma from which a single axon emanates. The axon comprised of alternating myelinated internodal regions (INRs) and unmyelinated nodal compartments, with 52 of the former and 52 of the latter. The INRs were further divided into internodal, juxtaparanodal, and paranodal regions with appropriate specifications (Devaux and Gow, 2008). The morphologic properties of the axon were as described in (Kolaric et al., 2013). The passive electric properties of the axon were based on collated data from corpus callosum axons and oligodendrocytes (Bakiri et al., 2011). Channel distribution followed historical data with voltage-gated sodium channels implemented at the node (Rasband and Trimmer, 2001) to facilitate saltatory conduction. Potassium channels were distributed as described in (Gordon et al., 1988) and passive leak channels were distributed internodally. Rate constants for voltage-gated channels are as described in (Brown and Hamann, 2014). Simulated action potentials were computed using backward Euler integration with a time step of 0.01 msec. We modified the myelin conductance (g_myl_) to reproduce the possible toxin-induced changes. If the modeling were performed at 37 degrees the action potentials would be smaller in amplitude, shorter in duration and the conduction velocity would be increased. However, changes in temperature would have no qualitative effect on the data and conclusions.

### Transmission electron microscopy

Optic nerves after obtaining a CAP response were fixed for at least 24 h at 4°C in 1.5% glutaraldehyde in 0.07 M phosphate buffer, pH 7.4. Fixed nerves were shortly washed in phosphate buffer and then in 0.1 M sodium cacodylate buffer. Nerves were then post-fixed with 1% osmium tetroxide/1.5% potassium ferricyanide, dehydrated in ethanol and embedded in EPON-812 resin. A segment containing the recorded nerve was cut and included in a down sided oriented capsule in which the nerve was situated in the lower surface. Semithin and ultrathin sections were obtained using a Reichert-Jung Ultracut E microtome. Sections were stained with uranyl acetate and lead citrate and viewed in a Jeol 1010 electron microscope (Electron Microscope core Facilities of the University of Barcelona). Once the images were obtained and to measure the distance between major lines inside the myelin sheath, we adjusted brightness and contrast to enhance the major lines and delimited an area to measure the gray intensity hence measure the distance between black and white peaks. By the use of ImageJ software (RRID:SCR_003070) we plotted the light intensity versus distance of the area delimited and transferred the information into Igor software to adjust a sinusoidal function which was analyzed with a Fourier transform to calculate the frequency, and thus obtaining the distance between peaks.

### Statistical analysis

Data from averages are always expressed as mean ± SEM. For statistical analysis we used an unpaired two-tailed Student’s *t* test, except in for the case of train stimulation, where we used a paired two-tailed Student’s *t* test (GraphPad InStat version 3.10, GraphPad software; RRID:SCR_000306). Significance levels were set at *p < 0.05 and **p < 0.01. Significance levels are displayed in the Tables 1, 2, where the number represents points of the mean values from area and amplitude, comparing *t*_-5_ with *t*_50_ and *t*_80_.

**Table 1.** Statistical significance for amplitude peak fractional decrease

Point	Data structure	Type of test	Power	Significance
*t*_-5_ vs *t*_50_				
1	Normal	Paired two-tailed *t* test	0	
2	Normal	Paired two-tailed *t* test	0.0405	*
3	Normal	Paired two-tailed *t* test	0.0107	**
4	Normal	Paired two-tailed *t* test	0.0072	**
5	Normal	Paired two-tailed *t* test	0.0094	**
6	Normal	Paired two-tailed *t* test	0.0155	*
7	Normal	Paired two-tailed *t* test	0.0304	*
8	Normal	Paired two-tailed *t* test	0.0097	**
9	Normal	Paired two-tailed *t* test	0.0824	
10	Normal	Paired two-tailed *t* test	0.0103	*
11	Normal	Paired two-tailed *t* test	0.0571	
12	Normal	Paired two-tailed *t* test	0.0209	*
13	Normal	Paired two-tailed *t* test	0.0744	
14	Normal	Paired two-tailed *t* test	0.0279	*
*t*_-5_ vs *t*_80_				
21	Normal	Paired two-tailed *t* test	0	
22	Normal	Paired two-tailed *t* test	0.0188	*
23	Normal	Paired two-tailed *t* test	0.007	**
24	Normal	Paired two-tailed *t* test	0.0064	**
25	Normal	Paired two-tailed *t* test	0.0202	*
26	Normal	Paired two-tailed *t* test	0.0306	*
27	Normal	Paired two-tailed *t* test	0.0322	*
28	Normal	Paired two-tailed *t* test	0.0284	*
29	Normal	Paired two-tailed *t* test	0.0673	
30	Normal	Paired two-tailed *t* test	0.0418	*
31	Normal	Paired two-tailed *t* test	0.0522	
32	Normal	Paired two-tailed *t* test	0.0457	*
33	Normal	Paired two-tailed *t* test	0.0693	
34	Normal	Paired two-tailed *t* test	0.0551	
35	Normal	Paired two-tailed *t* test	0.0866	
36	Normal	Paired two-tailed *t* test	0.0682	
37	Normal	Paired two-tailed *t* test	0.084	
38	Normal	Paired two-tailed *t* test	0.0872	
39	Normal	Paired two-tailed *t* test	0.0927	

Significance power of statistical analysis of paired two-tailed *t* test of the peak amplitude, comparing the first peak of the 100-Hz train stimulation to the rest 18 peaks. *p < 0.05; **p < 0.01. (*N* = 7 control, 11 toxin).

**Table 2.** Statistical significance for area peak fractional decrease

Point	Data structure	Type of test	Power	Significance
*t*_-5_ vs *t*_50_				
41	Normal	Paired two-tailed *t* test	0	
42	Normal	Paired two-tailed *t* test	0.0123	*
43	Normal	Paired two-tailed *t* test	0.0482	*
44	Normal	Paired two-tailed *t* test	0.0518	
45	Normal	Paired two-tailed *t* test	0.0304	*
46	Normal	Paired two-tailed *t* test	0.014	*
47	Normal	Paired two-tailed *t* test	0.0142	*
48	Normal	Paired two-tailed *t* test	0.0187	*
49	Normal	Paired two-tailed *t* test	0.0207	*
50	Normal	Paired two-tailed *t* test	0.0293	*
51	Normal	Paired two-tailed *t* test	0.0921	
52	Normal	Paired two-tailed *t* test	0.1104	
53	Normal	Paired two-tailed *t* test	0.0437	*
54	Normal	Paired two-tailed *t* test	0.0442	*
55	Normal	Paired two-tailed *t* test	0.0275	*
56	Normal	Paired two-tailed *t* test	0.0309	*
57	Normal	Paired two-tailed *t* test	0.0327	*
58	Normal	Paired two-tailed *t* test	0.0254	*
59	Normal	Paired two-tailed *t* test	0.0304	*
*t*_-5_ vs *t*_80_				
61	Normal	Paired two-tailed *t* test	0	
62	Normal	Paired two-tailed *t* test	0.055	
63	Normal	Paired two-tailed *t* test	0.0579	
64	Normal	Paired two-tailed *t* test	0.0435	*
65	Normal	Paired two-tailed *t* test	0.0276	*
66	Normal	Paired two-tailed *t* test	0.0223	*
67	Normal	Paired two-tailed *t* test	0.0348	*
68	Normal	Paired two-tailed *t* test	0.0362	*

Significance power of statistical analysis of paired two-tailed *t* test of the peak area, comparing the first peak of the 100 Hz train stimulation to the rest 18 peaks. *p < 0.05. (*N* = 7 control, 11 toxin).

## Results

### Induction of ATP release from the mouse optic nerve on exposure to ε-toxin

**Figure 2.**
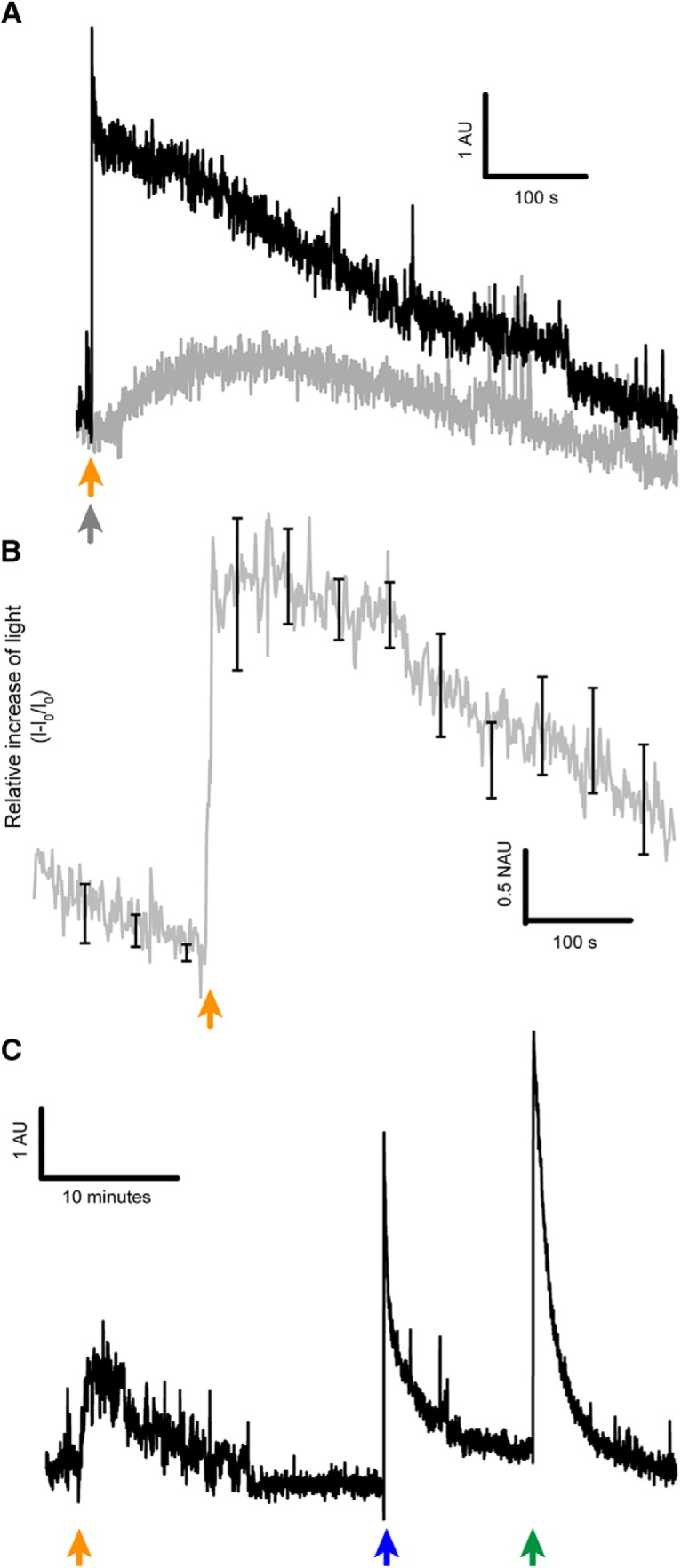
ATP release of the optic nerve on ε-toxin exposure. ***A***, Difference of light emission of ATP when optic nerves were treated with ε-toxin (black) or prototoxin (gray). Orange arrow indicates when ε-toxin is added and gray arrow when prototoxin is added. ***B***, Relative increase of light due to the release of ATP induced by ε-toxin (*n* = 4), mean values ± SEM in black. ***C***, Light emitted indicating ATP is released by the optic nerves once the ε-toxin is applied (orange arrow), ATP is also released when 500 µl of ultrapure water (mQ) was added (blue arrow) and with Triton X-100 at 0.02% (green arrow). Scale bar represented in each panel. AU, arbitrary units; NAU, normalized arbitrary units.

Using the luminescence reaction of luciferin and luciferase, we could continuously record the release of ATP from optic nerves suspended in a test tube. We observed that ε-toxin triggered a release of ATP and that this effect was specific, because prototoxin did not induce a significant release of ATP (Fig. 2*A*,*B*). Figure 2*C* shows that the decrease of osmotic tension by the addition of ultrapure water induced a large peak of ATP release, indicating that the ε-toxin did not deplete completely the content of ATP of the optic nerves. Moreover, a final addition of the detergent Triton X-100, permeabilized the optic nerves allowing the detection of the remaining ATP present in the optic nerve (Fig. 2*C*).

### Recording of CAPs in optic nerves

According to the above results, we looked for the effect of ε-toxin on the action potentials of myelinated fibers. Propagation of electrical signals in optic nerves occurs through CAPs (Evans et al., 2010). These signals reflect the addition of all action potentials conveyed by the optic nerve. To evaluate the pore mediated effect of the toxin over the optic nerve conduction, we submit the nerve to two opposite extreme conditions: low-frequency (0.03 Hz) and high-frequency (100 Hz) stimulations.

**Figure 3.**
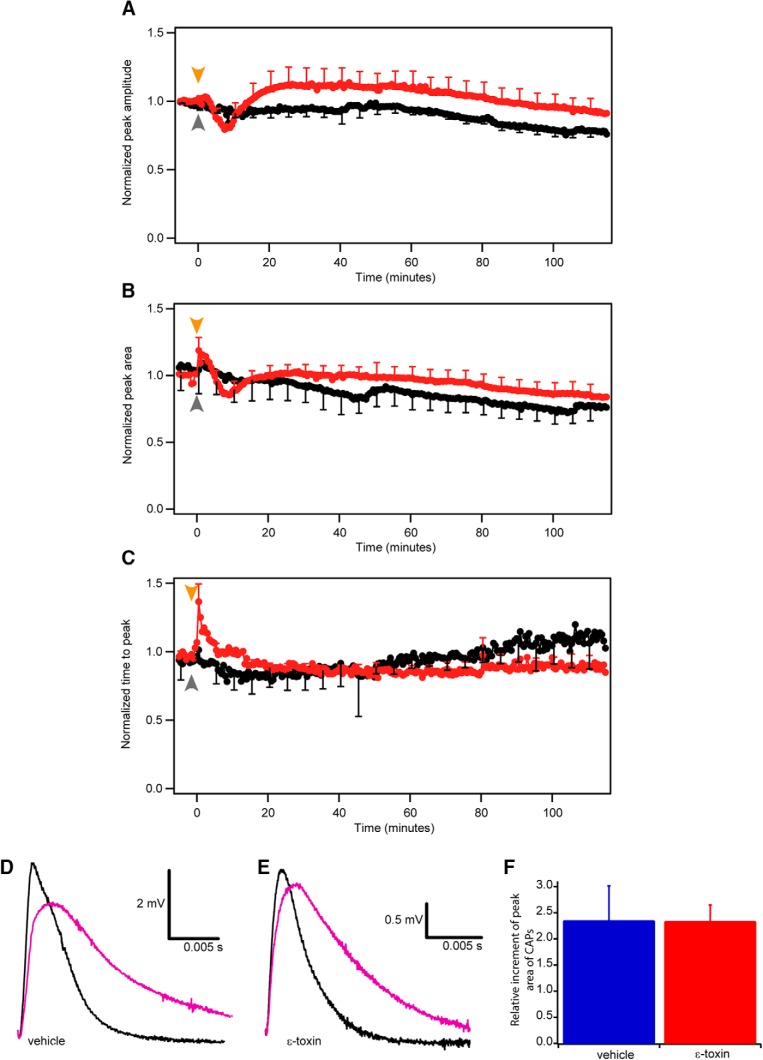
Time-course changes of the CAPs elicited at low frequency (0.03 Hz). Comparison of normalized peak amplitude (***A***), peak area (***B***), and time to peak (***C***) in control (black) and ε-toxin (red) conditions during 125 min. Normalization was done comparing the mean of the last nine CAPs before adding ε-toxin. Mean value ± SEM of control conditions (*n* = 7) and ε-toxin conditions (*n* = 11) are shown. Arrows represent when the vehicle (gray) or ε-toxin (orange) was added. After 115 min, 3,4-DAP is added to block potassium voltage-dependent channels present in the juxtaparanode. In nontoxin (***D***) and ε-toxin (***E***) conditions, the CAP before the addition of 3,4-DAP (black) and the CAP once 3,4-DAP is applied (purple). Scale bar represented in each panel. ***F***, Comparison of the area increment once 3,4-DAP is applied in control (blue, *n* = 4) and ε-toxin conditions (red, *n* = 11). Mean values ± SEM represented.

First, in 0.03 Hz stimulating condition and during the first 15 min of the incubation with the toxin, the amplitude of CAPs, the surface area delimited under CAPs and the latency measured as time to peak changed not significantly (*p* = 0.1945). During the remaining 125 min, at 0.03 Hz stimulation, the CAP profile did not vary along time, in control and after toxin treatment (Fig. 1*D*,*E*). Due to the variability of CAPs amplitude in individual nerves, we normalized the peak amplitude and we found that: optic nerves, treated with ε-toxin, had not significant higher peak amplitude than in control conditions. In toxin conditions the normalized amplitude was mostly above 1 while in control conditions the normalized amplitude was constantly decreasing below 1. Statistically analysis did not reveal significant differences (0.1713 < *p* < 0.999, control *n* = 7, toxin *n* = 11; Fig. 3*A*). The difference of the normalized peak area of CAPs along the 125 min, between control and toxin conditions was not significant (0.0657 < *p* < 0.9838, control *n* = 7, toxin *n* = 11; Fig. 3*B*). Similarly, the time to peak or latency measurements of CAPs from control and toxin conditions (Fig. 3*C*) did not show any significant difference (0.0661 < *p* < 0.9958, control *n* = 7, toxin *n* = 11). This suggests that the toxin does not alter profoundly the amplitude and area and does not affect the time to peak of the CAPs recorded at low stimulation rates.

#### ε-Toxin does not affect voltage-dependent potassium channels of the juxtaparanodes

The results obtained at a rate of stimulation of 0.03 Hz indicated that voltage-dependent channels of the Ranvier node were not affected after the action of ε-toxin. In fact, voltage-dependent K^+^ channels were still sensitive to blockers. In some experiments, after >115 min of incubation of the toxin, we added 3,4-DAP, a potassium channel blocker. After few minutes of the addition of 3,4-DAP, the CAPs were wider if compared with the condition of treatment with vehicle solution (Fig. 3*D*). The same change in the contour of CAPs was observed after 2 h under the action of ε-toxin (Fig. 3*E*). Our results show that the ε-toxin does not affect voltage-dependent potassium channels of the juxtaparanodes since the effect of 3,4-DAP is very similar in control conditions and in the presence of the toxin. We quantified the effect of 3,4-DAP by measuring the relative increment of the area once its added and we observed no significant difference (*p* = 0.982) comparing control and treated optic nerves (Fig. 3*F*).

### CAP in response to trains of stimuli

**Figure 4.**
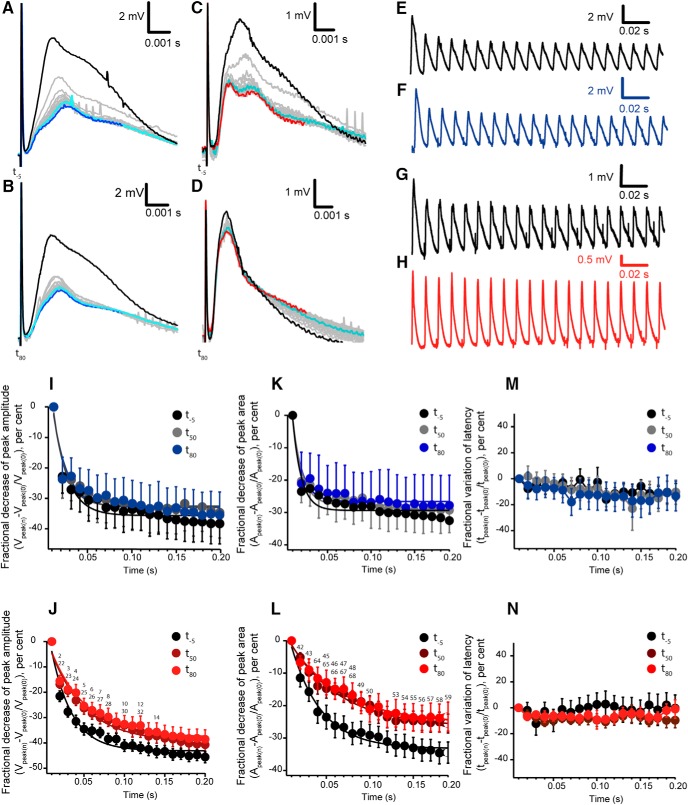
Analysis of CAPs elicited at high frequency (100 Hz). ***A***–***D***, The individual CAPs activated during the 200 ms train were synchronized at the time of delivering the stimulus pulse, showing an overall decrease. Representation of the CAP at the beginning of the train (black) to intermediate stages (light blue) and at the end of the train (dark blue) in control conditions at -5 min (*t_-5_*; ***A***) and at 80 min (*t_80_*; ***B***); gray traces correspond to the rest of CAPs. ***C***, ***D***, Trains before (*t_-5_*) and after adding (*t_80_*) the ε-toxin. Red traces correspond to the last CAP recorded in the train. In some traces, small transient spikes were recorded and are related to the interference of the peristaltic pump used for perfusion. Stimulus artifact was not removed. ***E***–***H***, Overall representation of the CAPs elicited by the 100 Hz stimulation in control conditions, to which only vehicle was added, at (***E***) *t*_-5_ and (***F***) *t*_80_. CAPs elicited by the 100 Hz stimulation in ε-toxin conditions at *t*_-5_ (***G***) and *t*_80_ (***H***) after the adding the ε-toxin. Stimulus artifact was eliminated in ***E***–***H***. ***I***, ***J***, Fractional decrease of peak amplitude in control conditions and with the treatment of ε-toxin at the three different train stimulations: *t*_-5_, *t*_50_, and *t*_80_. ***I***, Control conditions. ***J***, ε-toxin condition. Fractional decrease of the amplitude reflects the change of the amplitude of each of the 20 CAPs with respect to the first peak recorded when starting the train. Statistical significances are indicated by a number on the significance Table 1. ***K***, ***L***, Fractional decrease of peak area in control conditions and with the treatment of ε-toxin at the three different train stimulations: -5, 50, and 80 min. ***K***, Control condition. ***L***, ε-Toxin condition. Statistical significances are indicated by a number on the significance Table 2. ***M***, ***N***, Fractional variation of latency in control conditions and with the treatment of ε-toxin at the three different train stimulations *t*_-5_, *t*_50_, and *t*_80_. ***M***, Control conditions. ***N***, ε-Toxin condition. ***I***–***N***, Data are presented as mean values ± SEM; control (*n* = 7) and ε-toxin (*n* = 11).

To assess any change in passive properties of the nerve fibers, in between the 0.03 Hz stimuli, we applied three train stimulations of 100 Hz that lasted for 200 ms. The stimulation raised repetitive peak CAPs (Fig. 4*A-H*), where we analyzed their amplitude, area and time to peak. We observed a decrease of the size of the CAPs recorded. We quantified the decrease of the amplitude by calculating the fractional decrease in comparison of the first peak amplitude in the train. In other words, we quantified the percentage of amplitude decrease of each CAP with respect to the first CAP elicited during the train of stimuli and we adapted an exponential function to the decrease. In control conditions, the amplitude of the CAPs along the train decreases similarly between the three trains, one applied at -5 min (*t*_-5_) before adding the vehicle, and two activated at 50 min (*t*_50_) and 80 min (*t*_80_) after adding the vehicle (0.955 > *p* > 0.1316, *n* = 7; Fig. 4*I*). Single exponentials fitted the fractional decrease of the amplitude of CAPs, their time constant values were τ_(_*_t_*_-5)_ 16 ± 2.4 ms, τ_(_*_t_*_-50)_ 14 ± 1.9 ms and τ_(_*_t_*_-80)_ 16 ± 2.7 ms, with no significant difference between these values (1 > *p* > 0.1095, *n* = 7). When ε-toxin is added, a single decaying exponential is also found, as in controls. However, notice that there is a significant difference between the fractional decrease of the majority of peaks of the three trains (Fig. 4*J*). In Table 1, there is the correspondence between the numbers of the figure and the statistical significance. Their calculated time constants were: τ_(_*_t_*_-5)_ 25 ± 2.9 ms, τ_(_*_t_*_50)_ 42 ± 4.9 ms and τ_(_*_t_*_80)_ 43 ± 4.7 ms. There was a significant difference between the time constant before and after adding the toxin (*p* < 0.0001, *n* = 11). In summary, ε-toxin attenuates the decrease of the amplitude of CAPs during the 100 Hz train stimulation.

Considering the complex profile of the CAPs of the mouse optic nerve we also analyzed the variations of the area determined by each individual CAP of the train (Fig. 4*A-D*,*K-L* summarizes the results obtained) the fractional decrease in the area of CAPs during the train fitted to an exponential decay. In control conditions, where only vehicle was added, there was no change in the decrease when comparing their time constant τ_(_*_t_*_50)_ 11 ± 1.7 ms and τ_(_*_t_*_80)_ 11 ± 1.9 ms with respect to τ_(_*_t_*_-5)_ 10 ± 1.9 ms (0.3442 > *p* > 0.3198, *n* = 7; Fig. 4*K*). However, in ε-toxin conditions, the decrease in the area was attenuated after 50 and 80 min of action (Fig. 4*L*). The statistical significance between *t*_-5_, *t*_50_, and *t*_80_ are indicated in Table 2. Their corresponding values of time constant of the exponentials show significant differences between the three times, τ_(_*_t_*_-5)_ 34 ± 2.8 ms, τ_(_*_t_*_50)_ 57 ± 6.0 ms, and τ_(_*_t_*_80)_ 59 ± 5.9 ms (*p* < 0.0001, *n* = 11). Notice that the analysis of decrease of the area of CAPs is very similar with the results obtained analyzing the amplitudes. The action of ε-toxin on fiber excitability was tested measuring the time to peak of the CAPs; there was no significance difference in control (0.9892 > *p >* 0.2853, *n* = 7) and ε-toxin (0.9601 > *p >* 0.0825, *n* = 11) conditions (Fig. 4*M*,*N*). Hence, the toxin has an effect over the amplitude and area parameters; it attenuates the decrease of the CAPs during high-frequency stimulation.

### Computer simulations of action potential propagation in the optic nerve

**Figure 5.**
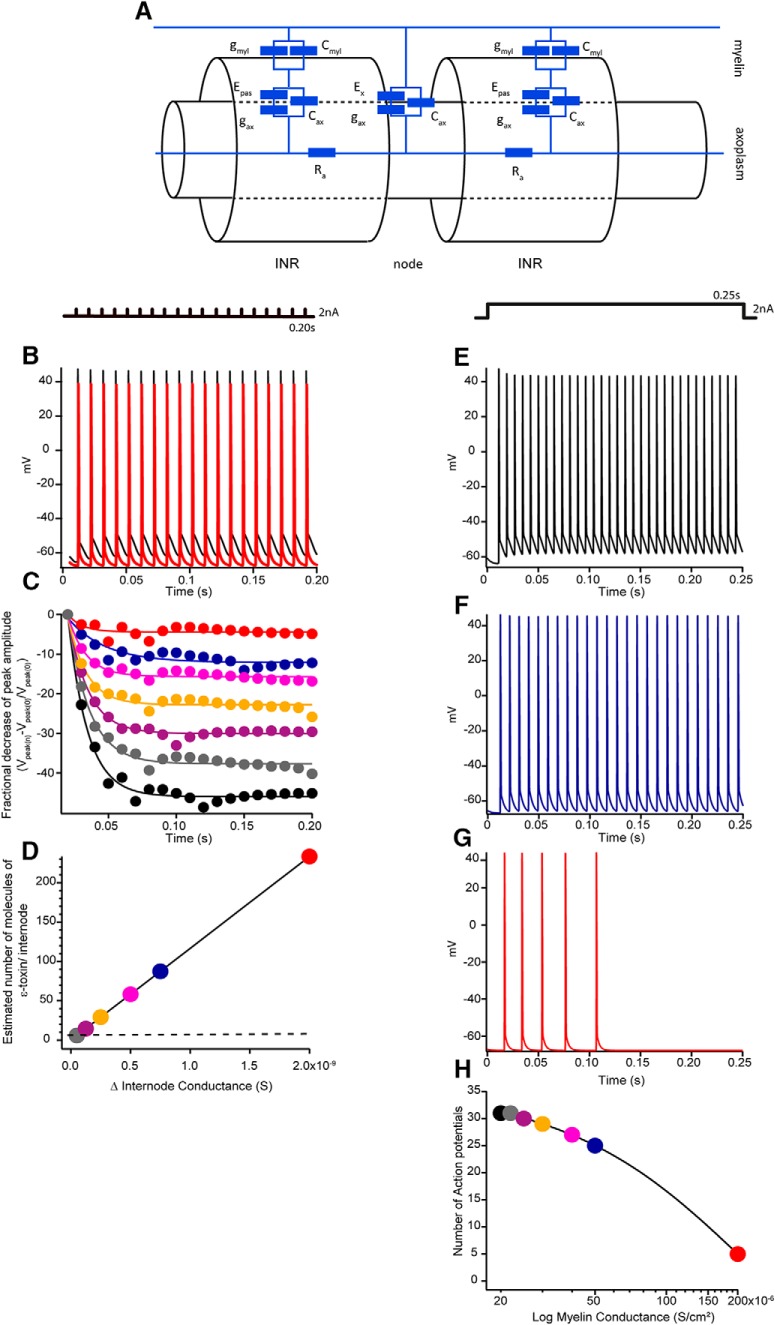
Computer simulation on a single myelinated fiber with the dimensions of the mouse optic nerve. ***A***, Equivalent circuit of the model corpus callosum axon, which has been adapted from existing models (Richardson et al., 2000; Devaux and Gow, 2008; Kolaric et al., 2013). The axon is divided into two regions, the node and internodal (INR). Nodal membrane potential is represented by E_x_ and intermodal potential by E_pas_. The nodal region expresses voltage-dependent conductances, as well as leak current (g_ax_) and capacitance (C_ax_). The axolemma underlying the myelin also has these properties (g_ax_ and C_ax_), but the myelin contributes an additional resistive (g_myl_) and capacitative barrier (C_myl_). The passive current across the membrane is the sum of g_myl_ and g_ax_. Axon resistance (R_a_) is constant throughout the model. Effect of the increasing g_myl_ on simulated action potentials during 100 Hz train. ***B***, Nineteen action potentials elicited in a 2 nA pulses train stimulation of 200 ms as represented above. In black, are action potentials of control conditions, with normal conductance (2 × 10^−5^ S/cm^2^) and in red, an increase of magnitude by 10 in conductance (2 × 10^−4^ S/cm^2^). ***C***, Representation of the fractional decrease of the peak amplitude of the 2-nA 100 Hz train stimuli at a different myelin conductance. Control conditions, 2 × 10^−5^ S/cm^2^ (black), 2.2 × 10^−5^ S/cm^2^ (gray), 2.5 × 10^−5^ S/cm^2^(purple), 3 × 10^−5^ S/cm^2^ (yellow), 4 × 10^−5^ S/cm^2^ (fuchsia), 5 × 10^−5^ S/cm^2^ (blue), and 2 × 10^−4^ S/cm^2^ (red). ***D***, Linear relationship between the increase of conductance of a single internode and the estimated number of molecules of the ε-toxin per internode. Few tens of molecules would increase the myelin conductance with a dramatic consequence on the amplitude decay as shown in ***C***. ***E***, Action potential triggered by a sustained 250 ms pulse of 2 nA, as shown above, at a control condition with a conductance, 2 × 10^−5^ S/cm^2^. ***F***, In blue, action potentials triggered at a conduction of 5 × 10^−5^ S/cm^2^, the frequency of firing is decreased. ***G***, In red, action potentials triggered at a conduction of 2 × 10^−4^ S/cm^2^, the frequency decreases until no action potentials can be elicited. ***H***, Plot of the calculated number of action potentials triggered versus myelin conductance. Code color as ***C***, ***D***. As the conductance of myelin increases, the number of action potentials elicited is decreased.

We tested the effect of ε-toxin on a modeled myelinated nerve fiber using Neuron 7.1 (Fig. 5*A*; see Materials and Methods for details). Figure 5*B* shows the action potentials generated in a single myelinated fiber when running the same train of stimuli that we applied experimentally (Fig. 5*B*, black traces). ε-Toxin is a pore forming toxin and it binds to myelin wrap, accordingly it decreases the electric resistance of myelin by the formation of pores. The increase of the myelin conductance (g_myl_) induces a change in the amplitude of the repetitive action potentials (Fig. 5*B*, red traces). The fractional decrease of the amplitude fits a decaying exponential function (Fig. 5*C*) similar to that measured in optic nerves (Fig. 4*I*,*J*). Interestingly, the increase of g_myl_ (2 × 10^−5^ S/cm^2^ – 2 × 10^−4^ S/cm^2^) induces a change in the shape of decaying curves. Discrete increases of g_myl_ attenuate the decreasing curves and at 2 × 10^−4^ S/cm^2^ the attenuation is completely abolished.

At present with the available methodology, it is extremely difficult to reliably record intracellular action potentials from single axons in the optic nerve (Gordon et al., 1988), due to the small diameter of axons. The median axon diameter in optic nerve is 0.7 μm, ranging from 0.09 to 2.58 μm (Allen et al., 2006). Alternatively the computational model allows us to explore what would be the effect of the toxin under current clamp conditions and to test the expected increase of g_myl_ due to the action of the toxin. Figure 5*E-G* show the autoregenerative action potentials when the fiber is set under a sustained pulse of 250 ms of 2 nA of current. In physiologic conditions, the optic nerve has firing frequencies over 100 Hz (Meister, 1996). Experimentally, we observed that an increase of the g_myl_ decreased the frequency of firing of the fiber. In control conditions of g_myl_ (2 × 10^−5^ S/cm^2^), the frequency of firing was 124/s (Fig. 5*E*) and the frequency decreased to 84/s (Fig. 5*F*) at a conductance of 5 × 10^−5^ S/cm^2^. Finally at 2 × 10^−4^ S/cm^2^, the frequency dropped to only firing 5 unevenly distributed action potentials in the first 100 ms of the pulse and afterward sent in a silent period and no more action potentials were elicited (Fig. 5*G*). The decay of the number of action potentials elicited during the increase of the conductance can be fitted with an exponential (Fig. 5*H*). Hence, when the conductance of myelin was increased, the frequency of action potentials elicited decreased which is not appreciable at first sight in Figure 5*F*, but is clearly represented in Figure 5*G*,*H*. It should be also noticed that the firing tends to be unevenly spaced at the higher conductance (Fig. 5*G*). In summary, the increase of g_myl_ alters the propagation of action potential in myelinated fibers. The simulation that we have done agrees with the fact that the pores made by the toxin increase the myelin conductance and alter the axonal conduction, making it less efficient.

### Electron microscopy

**Figure 6.**
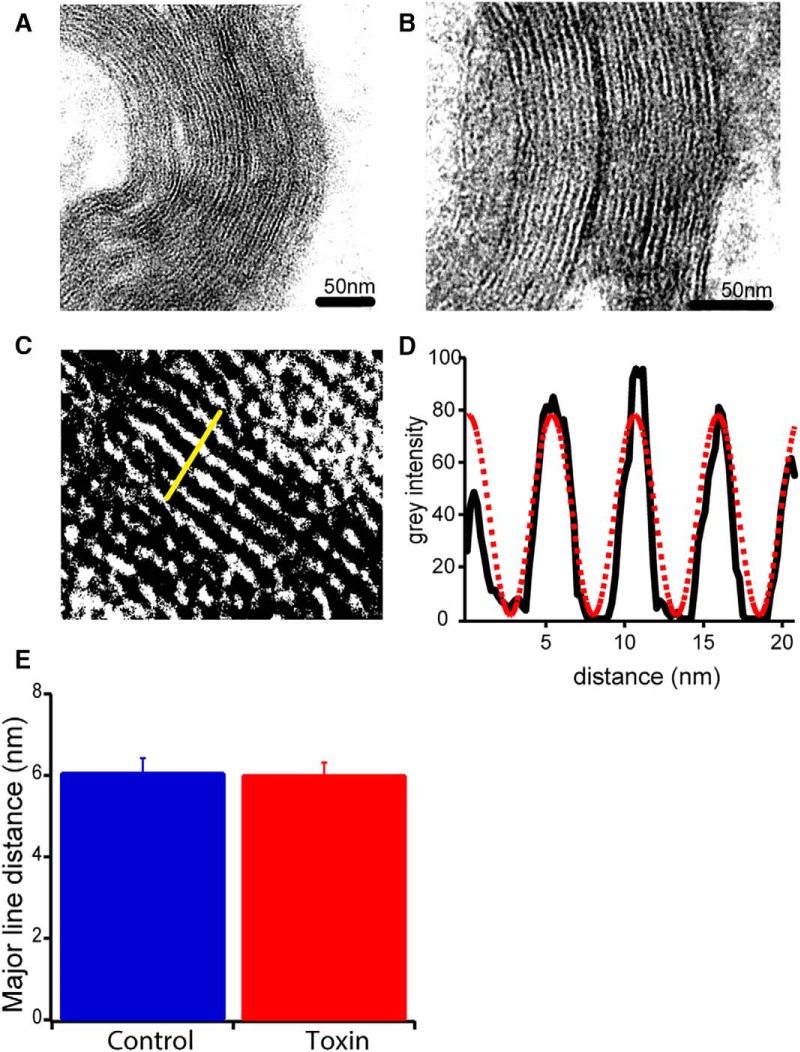
Electron microscopy of compact myelin from optic nerves. Electron microscopy images of myelin in (***A***) control conditions and (***B***) toxin conditions. Scale bar is represented in every panel. ***C***, Magnification over myelin layers to quantify distance on major lines. Contrast and brightness were modified to enhance the dense line of the myelin. Yellow bar (20 nm) represents the line through which the gray intensity was measured. ***D***, Graphic representation of gray intensity (black line) measured in ***C***. A sinusoidal function was fitted to calculate the distance between peaks (red dashed line); the distance between the dense lines was estimated after applying a Fourier transform. ***E***, The mean distance between major lines in control (blue; 30 images analyzed, *n* = 2 mice) and toxin (red; 30 images analyzed, *n* = 3 mice) conditions. No significant difference was found between the both means (*p* = 0.9062). Mean values ± SEM represented.

Complementary to the electrophysiological work, we wanted to see if the toxin causes structural changes in the optic nerve. Correlatively to electrophysiology, we proceeded with electron microscopy analyses of the structure. Visually, we could not observe any structural changes in the axons and in the myelin sheaths between control and toxin conditions (Fig. 6*A*,*B*). Moreover, we calculated the distance between the major lines on the myelin structure in control and toxin conditions. We adjusted the images to enhance the major lines and we selected areas to calculate the gray intensity (Fig. 6*C*). Fitting a sinusoidal function over the gray intensity and applying a Fourier transform (Fig. 6*D*), we measured the repetitive distance between the major lines formed by the myelin sheaths. The mean distances in control and toxin conditions were, respectively, 6.06 ± 0.36 nm (30 fibers, 2 animals) and 6.00 ± 0.30 nm (30 fibers, 3 animals). No significant difference was found between the two conditions (*p* = 0.906; Fig. 6*E*). Thus, we can conclude that the toxin does not affect the ultrastructure of myelin on short time exposure.

## Discussion

*C. perfringens* type B and D are important pathogenic factors in veterinary medicine because they induce enterotoxaemia in ruminants and have a strong economic impact in worldwide livestock (Freedman et al., 2016). The ε-toxin intoxication is characterized by brain edema and gut lesions in sheep or goats (Uzal and Songer, 2008; Bokori-Brown et al., 2011; Wioland et al., 2013). Acute intoxication of ε-toxin by direct inoculation of bacterial suspension into the duodenum produces the following neurologic symptoms: increased respiratory efforts, recumbency, paddling, bleating, convulsions, blindness, and opisthotonus (Uzal et al., 2004). Remarkably, blindness is a symptom related to our finding of ε-toxin action on the optic nerve conduction during high-frequency action potentials firing.

As indicated in the introduction, ε-toxin binds to myelin (Dorca-Arévalo et al., 2008). In fact, a protein present in the compact myelin (MAL, Myelin and Lymphocyte protein) has been identified to be a key element for the binding and activity of ε-toxin (Rumah et al., 2015). Our investigation was oriented to understand the acute effect of the toxin on the myelinated tracts of the central nervous system. We decided to use the optic nerve of mice as a model due to its easy accessibility and handling.

Because of the high structural similarity to aerolysin produced by *Aeromonas hydrophila*, ε-toxin is considered a pore forming toxin (Alves et al., 2014). ε-toxin exerts its action only on some specific cells, such as: MDCK, renal mpkCCDc_14_ collecting duct cells, and human leiomyoblastoma cells (Popoff, 2011) where it binds, inserts a portion of its polypeptide structure, oligomerizes and makes a heptameric pore in the plasma membrane of the target cells (Alves et al., 2014; Khalili et al., 2017). The result is cell permeabilization which may include nonselective ion diffusion and the release of small molecules from inside the cell (Petit et al., 2001; Lonchamp et al., 2010). Cell lines sensitive to the toxin exhibit swelling followed by mitochondrial disappearance, blebbing and membrane disruption (Popoff and Poulain, 2010). Moreover, renal mpkCCDc_14_ collecting duct cells treated with ε-toxin show a 75% depletion of ATP in 15 min, as well as, a dose-dependent reduction of the transepithelial electrical resistance (Chassin et al., 2007).

Optic nerves contain astrocytes, microglia, blood capillaries but mainly axons wrapped with myelin from the extensions of oligodendrocytes. Due to the fact that ε-toxin binds to myelin (Dorca-Arévalo et al., 2008; Wioland et al., 2015), we study the possibility that the ε-toxin would induce the release of ATP from the optic nerve.

In lipid planer bilayers, ε-toxin makes ionic channels with a single-channel conductance of 60 pS in 100 mM KCl, which represents general diffusion pores. The channels are slightly selective for anions (Petit et al., 2001; Nestorovich et al., 2010). In line with these previous observations, we observed a transient release of ATP from the optic nerves after the immediate (5 min) application of the toxin. The origin of this ATP release is most likely to be from oligodendrocytes, the most abundant cell type in optic nerve. Yet, the effect of the toxin over the oligodendrocytes is still in discussion (Linden et al., 2015; Wioland et al., 2015). It has been suggested that the toxin activates glutamate release which in turn increases the cytosolic calcium concentration to finally produce demyelination but not causing direct oligodendrocyte death (Wioland et al., 2015). Conversely, it is also claimed that the toxin causes direct oligodendrocyte death and in consequence, demyelination (Linden et al., 2015). It must be remarked that these studies have been done in primary cultured cells, whereas our study provides a new insight on ATP release on “*ex in vivo*” animal model.

While, ε-toxin has been described to induce a cerebral edema in animals (Finnie, 2004), no electrophysiological report has been claimed on the toxin effect on nerve conduction. Since it is known its capacity to cross the BBB (Freedman et al., 2016) and cause demyelination, we used the optic nerve model to reveal a possible effect of the toxin on myelinated fiber conduction. Considering our experiments lasted for approximately 2 h, we observed exclusively the acute effect of the toxin on the CAPs elicited by low or high rate stimulations.

ε-Toxin was activated before the experiments at a limited amount due to safety restrictions. Accordingly a closed circuit of perfusion for recording CAPs in isolated optic nerves was set up and characterized. Our results show that the reuse of a small volume of perfusion liquid, constantly gassed, in low rate of stimulation of optic nerve did not introduce a deep perturbation as only a moderate decrease of the amplitude and the area of CAPs was observed during the 2 h experiments. The profile of the CAPs that we obtained in our experiments were very similar to that shown in two recent papers in which the authors used the same mice strain C57BL/6J (Wang et al., 2012; Etxeberria et al., 2016) and different from those obtained using CD1 Swiss albino strain.

At low-rate stimulation, no remarkable change in the amplitude, area and latency of CAPs after the addition of ε-toxin was observed. Apparently, the ion channels in the juxtaparanode were not modified. At least in the case of Kv channels, the profile of CAPs after blocking them with 3,4-DAP was not different between control conditions and after the action of ε-toxin.

The most striking electrophysiological result that we obtained was the attenuation of the decrease of CAPs elicited by train stimulation. To understand this, in terms of the electric cellular components, we used a computer model of single mouse myelinated fiber of the optic nerve and we found that the increase of myelin conductance appropriately simulates the attenuation of the decrease of the CAPs recorded in optic nerves. We did not model the activity-dependent interstitial K^+^ accumulation that would surely arise at high-frequency stimulation. Equivalent simulations have shown that conduction block occurs at stimulation frequencies <100 Hz greater than those used in this paper (Brazhe et al., 2011).

In the model we increased myelin conductance as a consequence of the insertion of ε-toxin into myelin, presumably forming heptameric pores. An increase (Δ) of myelin conductance of a single internode of 5 × 10^−11^ S would be achieved approximately with a single pore with a conductance of that of the ε-toxin (6 × 10^−11^ S; Petit et al., 2001) which would correspond to the coordinated action of 7 molecules of ε-toxin (Fig. 5*D*, gray dot). The action of 90 molecules of ε-toxin will render an estimated Δ of conductance of 7.5 × 10^−10^ S/cm^2^ (Fig. 5*D*, blue dot) and would jump the g_myl_ to 5 × 10^−5^ S/cm^2^, which will attenuate the decrease of the amplitude of CAPs (Fig. 5*C*, blue dots). The increase of myelin conductance may have a pathophysiological significance. Physiologically, sustained stimulus can be found in the retina: the ON ganglionar neurons. Our computer simulations reveal that the spontaneous generations of action potentials during these prolonged excitation periods are very sensitive to the myelin conductance because its increase induces a reduction of the frequency of action potentials generated (Fig. 5*H*). We should expect, in these conditions of high myelin conductance, that the integration of visual information would arrive desynchronized to the thalamus. Therefore, our results relate the action of the toxin on myelin and link the effects of the toxin with the visual symptoms of infected ruminants.

We wondered whether the insertion of the toxin would be a first and early step of demyelination and if ε-toxin would disorganize the concentric structure of myelin. Electron microscopy analysis indicates that during the 2-h period investigated, there is no change in the repetitive structure of myelin of the optic nerves. Hence, we cannot state that the toxin causes a disorganization of the myelin on acute exposure.

In addition to the effect of ε-toxin in livestock, further speculation relates the effect of the toxin in humans with MS (Rumah et al., 2013). MS is a neuroinflammatory disease of the central nervous system with an important participation of the immune system (Lassmann and Bradl, 2017). The main pathologic hallmark of MS patients consists of multifocal lesions, plaques, in the white matter. More than 85% of MS patients follow a relapsing and remitting model, which consists of episodes of deterioration of neurologic function followed by periods of recovery (McDonald et al., 2001). The etiology of MS is elusive and different hypothesis have been raised. Genetic and environmental factors have been suggested to play a role on the onset and development of the disease (Tao et al., 2017). A key, but not unique, feature of the disease is demyelination, where the conduction of neuronal signals would fail. Likewise, our results do not contradict a possible action of ε-toxin, in relation to MS.

To conclude, our study provides a new insight of the acute effects of ε-toxin over the central nervous system using the optic nerve as an experimental model. In fact, through magnetic resonance imaging, the optic nerve is explored to record the remyelination of the central nervous system after a relapse event in MS patients (Frohman et al., 2005; Balcer, 2006; Sakai et al., 2011). Hence, if the toxin has an effect over the optic nerve demyelination, it could be related to the onset of impaired vision in relapse events in MS. Although, many more studies should be aimed to understand the effects of the toxin on oligodendrocytes and the possible interaction of MAL protein with ε-toxin in a manner that we can further decipher the cellular effects of the toxin.

## Synthesis

Reviewing Editor: Alfonso Araque, University of Minnesota Decisions are customarily a result of the Reviewing Editor and the peer reviewers coming together and discussing their recommendations until a consensus is reached. When revisions are invited, a fact-based synthesis statement explaining their decision and outlining what is needed to prepare a revision will be listed below. The following reviewer(s) agreed to reveal their identity: Jordi Molgo, Francisco Uzal.

The reviewers have found some interest on the manuscript. However, they have expressed some concerns that need to be properly addressed.

Reviewer #1

In this article the action of epsilon toxin (ϵ-toxin) from Clostridium perfringens has been studied. Authors show that the toxin induced ATP release from isolated mouse myelinated optic nerves. Also, the toxin is reported to affect compound action potentials (CAPs) from isolated nerves, particularly during high frequency nerve stimulation (100 Hz). In fact the ϵ-toxin attenuated the decrease of CAP amplitude. Computer modelling mimicked ϵ-toxin experimental results, when increasing the myelin conductance, interpreted as resulting from the pore forming activity of the toxin. The ultrastructure of myelin on the time-frame investigated was not modified by the ϵ-toxin. The acute functional impact of the ϵ-toxin is related to alterations in nerve CAP propagation in myelinated axons from optic nerves. The possible participation of ϵ-toxin in the etiology of MS is discussed along the manuscript.

General comments.

This article in places is difficult to read as indicated in the specific comments here below. One has the impression that the authors are sending a preliminary version, instead of a final submission version. Some English terms used are difficult to understand.

Specific comments

1.Title of article: Please indicate Clostridium perfringens ϵ-toxin, since there are other ϵ-toxins from venomous animals in the animal phyla.

2.Page 5, line 72, the term "supported" is better than the term "sustained"

3.Page 5, line 78 "we wanted to undercover??

4. In the Materials and Methods section Page 8, line 129; revise the sentence since it's not clear

5. Page 8, line 123, indicate at which temperature recordings were performed

6. Page 9, line 153, Indicate the pH of the solution containing 3,4-DAP .

7. Page 9, line 151, it is not clear how the 200 nanometer concentration of ϵ-toxin was chosen. Please explain

8. Page 10, line 170, it should be : "alternating internodals" instead of "intermodal"?

9. Page 11, lines 214-216 (include reference(s) for the sentence.

10. Page 12, lines 214-219, In the Results section authors should start with the results obtained, therefore lines 214-219 should be moved either to the Introduction or Discussion. The comparison with other cell models like mpkCCDc14 cell line is certainly for the discussion.

11. In Figure 2B indicate the vertical calibration 0.5 ?

12. Page 12, lines 229-234 should go to the discussion

13. Page 13, Line 245, "should read: During the remaining 125 minutes, at 003 Hz stimulation, the CAP profile did not vary along time, in control and after toxin treatment.

14. Page 13, lines 253, also 256 what is meant by "usually" the statistics have values, just indicate the values, the use of this kind of terms make the manuscript unclear and wooly.

15. Page 14, Line 273, subheading "Recordings of train stimuli" what the authors are recording are: "CAP in response to trains of stimuli". Please amend.

16. Page 14, line 281, it should be "during the train of stimuli"

17. Page 15, Line 299, it should be "the fractional decrease in the area of CAPs during the train fitted to an exponential decay"

18. Page 16, line 313, in the modelling performed: what would be the effect of temperature i.e. the recordings were performed at room temperature? How the modelling will do at normal mammalian temperature of 37{degree sign}C (Q10 of 3.4?

19. Page 16, 326-327, add to the assertion " At present with the available methodology, it is not possible to electrically clamp a single nerve fibre isolated from the optic nerve", due to the small diameter of axons. Add some references and values of single axon diameter.

20. Page 17, line 358, it should be " the major lines formed by the myelin sheaths"

21. In the Discussion, the limitations of the modelling used should be outlined. For instance, how activity-dependent potassium accumulation in the extracellular space at high frequency stimulation (100 Hz) could modify the excitability properties of myelinated axons? In view of the small diameter of optic myelinated nerve axons (07-1 micrometer), this effect is likely important.

22. Finally, the present manuscript should also be revised in order to avoid duplication and repetition of information in the Introduction and Discussion.

Reviewer #2

Interesting paper. A few comments that might help the authors improve this Ms:

Line 7: ETX is synhesized as a "less" active toxin, not a "non-active" toxin. Please claridy

Abstract in general: something has to be said about the role of ETX in animal disease.The link to MS is really weak and definitely to proved; please clarify that.

Introduction

Again, ETX is known and proven to be responsible for neurologic and respiratory disease in several ruminant species and of course in multiple laboratory animals (rats, mice among others). The possible role in the development of MS has been suggested but never proved. All this has to be clearly stated in the introduction to give the reader a truthful and fair representation of what is known about the role of this toxin.

Line 51. Produced as a low toxicity or something like that

Recombinant epsilon tooxin: how was the toxiciy measured? This should be tested either ina animal model (mouse)or cells.

How many optic nerves were used for each experiment? This has to be stated in each case.

Lines 425-427. Not really. Your only conclusion is that there was no structural change in the structure of myelin in 2 hours. You can not conclude that more time will produce changes. It may never happen. Please modify accordingly.

Line 426. Why limited access to ETX? This is really not acceptable.

Discussion in general. You need to discuss the relevance of your findings for disease in natural hosts (ruminants). For instance, can your findings explain the blindness observed in sheep with type DD ennterotoxemia? This should be clearly separated from your speculation on the possible role of this toxin in neuritis of humans with MS.

In summary, my opinion is that the authors are stretching their conclusions. The results are interesting and potentially important to understand the mechanism of action of ETX, but they should be evaluated based on known knowledge (i.e. the role of ETX on animal disease). While it is OK to speculate a little on the potential role of ETX in MS, throughout the paper it has to be made clear that this is just that: speculation.
